# Next generation sequencing data of a defined microbial mock community

**DOI:** 10.1038/sdata.2016.81

**Published:** 2016-09-27

**Authors:** Esther Singer, Bill Andreopoulos, Robert M. Bowers, Janey Lee, Shweta Deshpande, Jennifer Chiniquy, Doina Ciobanu, Hans-Peter Klenk, Matthew Zane, Christopher Daum, Alicia Clum, Jan-Fang Cheng, Alex Copeland, Tanja Woyke

**Affiliations:** 1 DOE Joint Genome Institute, Walnut Creek, California 94598, USA; 2 Newcastle University, Newcastle upon Tyne, NE1 7RU, UK

**Keywords:** Next-generation sequencing, Microbial communities, DNA sequencing, Metagenomics

## Abstract

Generating sequence data of a defined community composed of organisms with complete reference genomes is indispensable for the benchmarking of new genome sequence analysis methods, including assembly and binning tools. Moreover the validation of new sequencing library protocols and platforms to assess critical components such as sequencing errors and biases relies on such datasets. We here report the next generation metagenomic sequence data of a defined mock community (**M**ock **B**acteria **AR**chaea **C**ommunity; MBARC-26), composed of 23 bacterial and 3 archaeal strains with finished genomes. These strains span 10 phyla and 14 classes, a range of GC contents, genome sizes, repeat content and encompass a diverse abundance profile. Short read Illumina and long-read PacBio SMRT sequences of this mock community are described. These data represent a valuable resource for the scientific community, enabling extensive benchmarking and comparative evaluation of bioinformatics tools without the need to simulate data. As such, these data can aid in improving our current sequence data analysis toolkit and spur interest in the development of new tools.

## Background & Summary

By definition, benchmark studies aim to provide standards that can be used to evaluate the performance of a process. The field of nucleic acid sequencing and sequence data processing has witnessed immense developments towards optimizing the balance of sequencing cost, precision and overall applicability to real-world questions. This progress has routinely relied on experimental setups of defined nature to critically rate novel approaches. In recent years, mock communities have been assisting in a variety of laboratory and computational test experiments, which resulted in quantitative and qualitative evaluation of corresponding studied methods. For example, mock communities were generated for the comparison of DNA extraction methods^1–3^, for the development of a dual-index sequencing and curation pipeline for Illumina MiSeq generated amplicon sequence data^4–8^, and to evaluate the Ion Torrent sequencing platform for gene-targeted studies^9,10^. Similarly, Pabinger *et al.*^11–13^ used a mock community to benchmark MEMOSys, a web-based platform for metabolic models. The jumpstart consortium human microbiome project (HMP) data generation working group established a standardized protocol for ensuring high throughput consistency of 16S rRNA gene amplification and sequencing protocols by implementing a synthetic mock community of 21 known organisms, before finalizing their HMP 16S 454 protocol^14–16^. The HMP DNA and sequence data resources have not only enabled comprehensive characterization of the human microbiota, *e.g.*^17–19^, but also the use and development of a variety of advanced analysis tools. For example, chimera screening tools UCHIME and Chimera Slayer^1,3^, the OTU construction pipeline UPARSE^4,6–8^, and fine-tuned workflows for amplicon gene studies^9^ used HMP data generated from mock communities.

**Figure 1:**
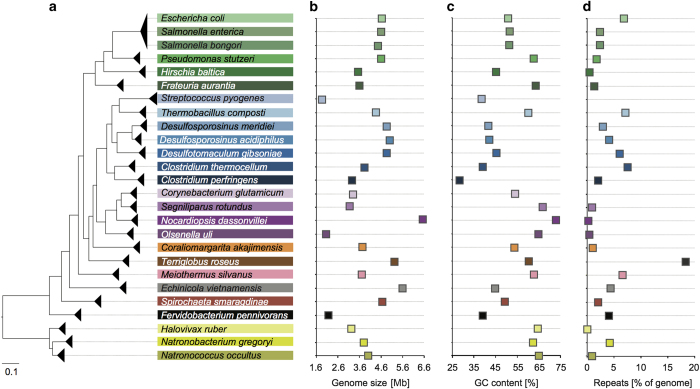
Characteristics of MBARC-26 community. Community members display diversity in phylogenetic distribution and relatedness (**a**), genome size (**b**), GC content (**c**), and repeat content normalized by genome size (**d**). Shades of the same color in (**a**) denote the same phylum association: Green—*Proteobacteria*, blue—*Actinobacteria*, purple—*Firmicutes*, yellow—*Euryarchaeota*.

**Figure 2:**
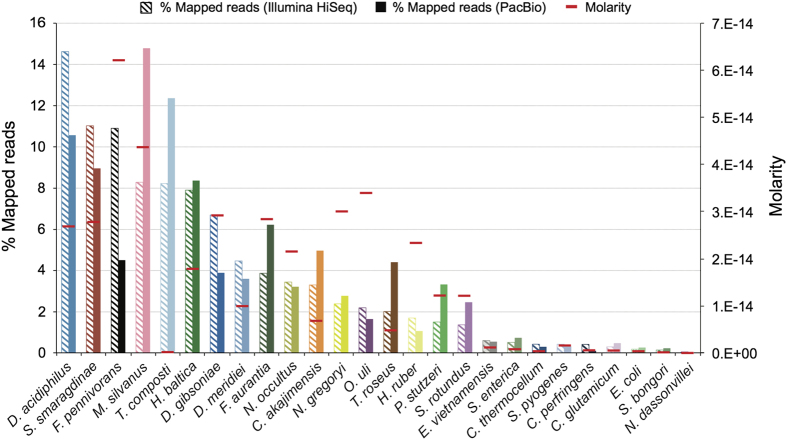
MBARC-26 community composition and relative abundance distribution, as based on Illumina and PacBio read mapping and mean DNA molarity. Mock community members are grouped and arranged in order of % mapped sequences (Illumina). The observed discrepancy between molarity and % mapped PacBio and Illumina sequences in *T. composti* is likely due to contamination as *T. composti* was previously found to occur as laboratory contaminant in various shotgun metagenome datasets (unpublished data). The smaller discrepancies are expected due to DNA quantification spreads and platform biases. Colors denote phylum association as defined in Fig. 1.

**Figure 3:**
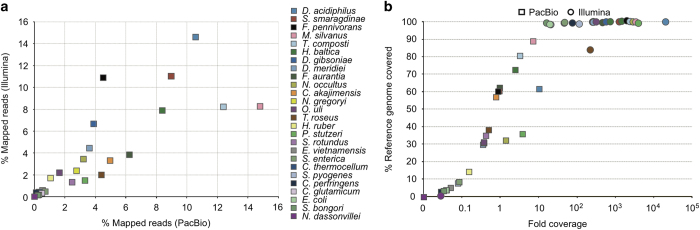
Quantitative comparison of MBARC-26 Illumina and PacBio shotgun sequence datasets. (**a**) Community representation according to % mapped sequences for each mock community member in the PacBio (*x*-axis) and Illumina (*y*-axis) shotgun sequence datasets. (**b**) Percent chromosome coverage and fold coverage of each mock community genome by sequencing platform using unassembled sequences. Colors denote phylum association as defined in Fig. 1.

In contrast to the HMP mock, the synthetic community described here, MBARC-26 (**M**ock **B**acteria **AR**chaea **C**ommunity), is composed of organisms isolated from heterogeneous soil and aquatic environments as well as derived from human, bovine and frog (Table 1). MBARC-26 consists of 23 bacterial and 3 archaeal strains, belonging to the phyla *Acidobacteria*, *Actinobacteria, Bacteroidetes, Deinococcus-Thermus, Firmicutes, (Alpha-* and *Gamma-*)*Proteobacteria, Spirochaetes, Thermotogae, Verrucomicrobia* and *Euryarchaeota*. Genome sizes span 1.8–6.5 Mbp, GC contents vary between 28.4–72.7%, and repeat content ranges from 0–18.3% (Fig. 1, Table 1). All genomes are available as finished genome sequences in GenBank (Table 1). MBARC-26 DNA was shotgun sequenced on Illumina HiSeq 2000 and PacBio RSII sequencing platforms (Table 2). We provide detailed descriptions of organism characteristics (Table 1), sample processing, including DNA extraction and quantification, sequencing library creation, and sequencing procedures (Table 2). Data statistics encompass sequencing throughput characteristics (Table 2), community structure according to read mapping to reference genomes and according to molarity (Fig. 2, Supplementary Table 1, Supplementary Fig. 1), quantitative comparison between Illumina and PacBio datasets (Fig. 3a, Table 1, Supplementary Figs 2 and 3), % genome coverage and fold coverage by sequencing platform (Fig. 3b), and GC content analysis (Supplementary Fig. 3). Due to inherent sequencing technology differences^11,13^, these two datasets are characterized by platform-, run mode-, and chemistry-specific read length, data throughput, GC and amplification bias, and error rate. We point out that our quantitative results are directly correlated to the respective sample preparation and sequencing methods used, as these have been shown to critically affect community representation^14,20^.

To date, several studies already utilized MBARC-26 and took advantage of its purposefully selected characteristics. Availability of complete reference genomes and relative abundance spread of individual constituents enabled determining lower limits of various metagenome library preparation protocols^14^. MBARC-26 was also used to develop a new full-length 16S rRNA gene amplicon sequencing protocol called PhyloTags^17^ and allowed for quantitative comparison of amplicon to shotgun sequence data and bias evaluation associated with GC content. Using the MBARC-26 Illumina metagenome dataset and corresponding single-cell sequence data Bremges *et al.* developed MeCorS, a metagenome-enabled single-cell read correction tool^21^. To further encourage the use of this mock community, we report the release of molarity and shotgun sequence datasets of MBARC-26.

Perpetual community efforts to develop improved DNA sequence analysis software with various applications for shotgun sequence data requires standardized and well-characterized data for benchmark experiments. MBARC-26 was validated according to the specific sample processing tools using a variety of commonly used quality control methods, is accompanied by data statistics, and meant to enable method development and evaluation while enabling reproducibility of research findings.

## Methods

These methods are expanded from descriptions in our previous work^17^.

### Cultivation and DNA extraction

DNA from *Escherichia coli, Salmonella bongori, Salmonella enterica, Clostridium perfringens, Clostridium thermocellum* and *Streptococcus pyogenes* was purchased from the American Type- Culture Collection (ATCC, Manassas, VA, USA). DNA from *Fervidobacterium pennivorans, Thermobacillus composti* and *Corynebacterium glutamicum* was extracted using phenol–chloroform extraction, as described in (ref. 22). DNA from *Desulfosporosinus acidiphilus, Desulfosporosinus meridiei, Desulfotomaculum gibsoniae, Echinicola vietnamensis, Frateuria aurantia, Natronococcus occultus, Olsenella uli* and *Terriglobus roseus* was isolated using the Jetflex Genomic DNA Purification Kit (Genomed GmbH, Loehne, Germany). DNA from *Hirschia baltica* was extracted using the Blood and Cell Culture DNA Maxi Kit (Qiagen, Valencia, CA, USA). DNA from *Meiothermus silvanus, Nocardiopsis dassonvillei* and *Segniliparus rotundus* was extracted using the Qiagen Genomic 500 DNA Kit (Qiagen, Hilden, Germany). DNA from *Pseudomonas stutzeri* was isolated using the Wizard Genomic DNA Purification Kit (Promega Corp., Madison, WI, USA). DNA from *Coraliomargarita akajimensis, Halovivax ruber, Natronobacterium gregoryi* and *Spirochaeta smaragdinae* was extracted using the Masterpure Gram-Positive DNA Purification Kit (Epicentre, Madison, WI, USA). All DNA extracts were quantified using the PicoGreen assay and the Qubit 2.0 fluorometer (Invitrogen, Carlsbad, CA, USA) (Supplementary Fig. 1). Each sample was quantified in quadruplicate. Samples were pooled at varying ratios to generate the mock community (Fig. 2, Supplementary Table 1).

### Library creation and sequencing

For Illumina library creation, 100 ng of genomic DNA of MBARC-26, brought up to a total of 100 μl using TE, was sheared to 300 bp using the Covaris LE200 (Covaris, Inc., Woburn, MA, USA) and size-selected using AMPure XP beads (Beckman Coulter, Brea, CA, USA): 60 μl of beads were added to 100 μl of sample. The sample was then incubated at room temperature (RT) for 5 min. Beads were pelleted using a magnetic particle concentrator (MPC) (Thermo Fisher Scientific, South San Francisco, CA, USA) until liquid was clear. The supernatant was removed and transferred to a new tube. 30 μl of AMPure XP beads were then added for the second bead size selection. The mixture was pulse vortexed, quickly spun and incubated at RT for 5 min. Beads were pelleted using a magnetic particle concentrator (MPC) (Thermo Fisher Scientific, South San Francisco, CA, USA) until liquid was clear. The supernatant was then discarded without disturbing the beads and 200 μl of freshly prepared 75% ethanol (EtOH) was added, followed by a 30 s incubation to wash the beads. EtOH was discarded before the wash step with EtOH was repeated for a total of two washes. Afterwards, the sample was placed on a thermocycler (Eppendorf, Hamburg, Germany) with the lid open and incubated at 37 °C until the beads were dry and residual EtOH had evaporated. The beads were re-suspended in 53 μl of EB buffer (Qiagen, Redwood City, CA, USA), vortexed, quickly spun and incubated at RT for 1 min. Beads were pelleted using an MPC until liquid was clear (Thermo Fisher Scientific, South San Francisco, CA, USA). 50 μl of supernatant was then transferred to a new tube. The DNA fragment size was assessed using the Agilent Bioanalyzer 2100 High Sensitivity Kit (Agilent Technologies, Palo Alto, CA, USA) before proceeding to end repair.

The fragments were treated with the Kapa Library Preparation Kit ORIGIN (Kapa Biosystems, Wilmington, MA, USA) for the following steps: For end-repair 26 μl MilliQ water, 9 μl 10X End Repair Buffer, and 5 μl End Repair Enzyme were combined in a 1.5 ml tube. The cocktail was vortexed and quickly spun, then stored on ice. 40 μl of End Repair cocktail was added to the 50 μl DNA sample. The mixture was vortexed and quickly spun, before incubation at 30 °C for 30 min in a thermocycler (Eppendorf, Hamburg, Germany). After incubation, 126 μl of AMPure XP beads (Beckman Coulter, Brea, CA, USA) were added to 90 μl of End Repair sample, pulse vortexed, quickly spun, and incubated at RT for 5 min. Beads were pelleted using a MPC until liquid was clear. The supernatant was then discarded without disturbing the beads. The beads were washed twice with 200 μl of freshly prepared 75% EtOH with an incubation time of 30 s. After washing, the sample was incubated at 37 °C in a thermocycler with the lid open until residual EtOH had evaporated. For DNA elution, 17.5 μl of EB buffer was added. The sample was vortexed, quickly spun, and incubated at RT for 1 min, before beads were pelleted on a MPC. 15 μl of supernatant was then transferred to a new tube.

For A-tailing, 9 μl of MilliQ water, 3 μl of 10X A-Tailing Buffer and 3 μl of A-Tailing Enzyme were combined in this order in a 1.5 ml tube. The cocktail was vortexed and quickly spun. 15 μl of the A-Tailing cocktail was added to the 15 μl sample. The mixture was vortexed and quickly spun. The samples were then incubated in a thermocycler at 30 °C for 30 min, followed by 5 min at 70 °C.

Adaptor ligation was immediately performed thereafter: 9 μl of 5X Ligation Buffer and 5 μl of ligase were combined in a 1.5 ml tube, vortexed and spun. The mixture was pulse vortexed and quickly spun. 14 μl of adaptor ligation cocktail were added to the 30 μl sample, before 1 μl of 18 μM adaptor was added to the ligation mixture for a final concentration of 400 nM. The mixture was incubated in a thermocycler at 20 °C for 15 min.

After adaptor ligation, 5 μl of EB Buffer was added to 45 μl of adaptor-ligated sample. The sample was size-selected and washed twice with 45 μl of AMPure XP beads as described previously. After the first clean-up step, the sample was eluted with 52 μl of EB Buffer and 45 μl of supernatant was transferred to a clean tube. After the second clean-up step, the sample was eluted with 25 μl of EB Buffer. 23 μl of supernatant was transferred to a clean tube. The sample was quality-controlled and quantified using an Agilent Bioanalyzer 2100 High Sensitivity Kit.

The prepared Illumina library was further quantified by using the Kapa Biosystems next-generation sequencing library qPCR kit according to the manufacturer’s guidelines (Kapa Biosystems, Wilmington, MA, USA). The amplification products were run on a Roche LightCycler 480 real-time PCR instrument for quantification (Roche Holding AG, Basel, Switzerland). The quantified library was then prepared for sequencing on the Illumina HiSeq sequencing platform (Illumina, Inc., San Diego, CA, USA). First, the TruSeq paired-end cluster kit, v3, and Illumina’s cBot instrument were used to generate a clustered flowcell for sequencing (Illumina, Inc., San Diego, CA, USA). Sequencing of the flowcell was performed on the Illumina HiSeq2000 sequencer using a TruSeq SBS sequencing kit 200 cycles, v3, following a 2x150 indexed run recipe (Illumina, Inc., San Diego, CA, USA) (Table 2). This resulted in 355,875,608 raw reads.

For PacBio library creation, 5 μg of gDNA was sheared using a Covaris LE220 to generate 2 kb fragments (Covaris, Inc., Woburn, MA, USA). The sheared DNA fragments were then prepared according to the SMRTbell template preparation kit guidelines (Pacific Biosciences, Menlo Park, CA, USA). Briefly, DNA fragments were treated with DNA damage repair mix, end-repaired, and 5’ phosphorylated. PacBio hairpin adapters were then ligated to the fragments to create SMRTbell template for sequencing. The SMRTbell templates were purified using exonuclease treatments and size-selected using AMPure PB beads (Pacific Biosciences, Menlo Park, CA, USA) (Table 2).

Sequencing primers were annealed and v. P4 sequencing polymerase was bound to the SMRTbell templates. The prepared SMRTbell template libraries were then sequenced on a Pacific Biosciences RSII sequencer using v. C2 chemistry and 1x120 min sequencing movie run times (Pacific Biosciences, Menlo Park, CA, USA). This resulted in 300,584 raw reads (Table 2).

### Sequence QC

Illumina shotgun reads were filtered using BBDuk (filterk=27, trimk=27; http://jgi.doe.gov/data-and-tools/bb-tools/) to remove Illumina adapters, known Illumina artifacts, phiX, and to quality-trim both ends to Q12. Resulting reads containing more than one ‘N’, or with quality scores (before trimming) averaging less than 8 over the read, or length under 40 bp after trimming, were discarded. Remaining reads were mapped to a masked version of human HG19, dog, cat, and mouse with BBMap (http://jgi.doe.gov/data-and-tools/bb-tools/), discarding all hits exceeding 93% identity. This resulted in 347,963,988 filtered reads with average insert size of 219±43 bp.

Quality filtering and error correction of PacBio sequences was performed using the RS_ReadsOfInsert protocol v. 2.3.0 in SMRT Portal (minimum subread length: 50 bp; minimum read quality: 75%). This resulted in 53,654 quality-filtered subreads with average read length of 1,041±576 bp.

### Mapping, repeat regions, and phylogenetic tree construction

High quality Illumina and PacBio sequences were mapped to their bacterial and archaeal reference genomes using BBMap with parameters bbmap.sh, ambig=toss (Illumina) and mapPacBio.sh, ambig=toss (PacBio), respectively. Numbers of mapped sequences were normalized to the respective whole genome and chromosome lengths of reference organisms (Supplementary Table 1). Unmapped sequences amounted to 2,105 (3.92%) and 3,777 (7.04%) PacBio sequences, when mapped against genome and chromosome references, respectively. In the Illumina dataset, 8,981,844 (2.58%) and 18,088,260 (5.20%) Illumina sequences remained unmapped, when mapped against genome and chromosome reference, respectively. Repeat regions reported here were retrieved from NCBI GenBank^23^ on May 16, 2016. They include tandem, inverted, flanking, terminal, direct and dispersed repeat types. For phylogenetic tree construction, full-length 16S rRNA gene sequences were aligned using the SINA aligner^24^ including 10 neighbors at 95% minimum identity for classification against the SILVA, RDP, greengenes, LTP, and EMBL databases^25^. The alignment was masked using the SILVA-compatible 1,349 Lane mask^26^. Tree construction was performed using FastTree^27^.

## Data Records

Filtered shotgun sequences generated on the Illumina and PacBio platforms are publically available through NCBI (Data Citation 1 and Data Citation 2).

## Technical Validation

To assess the quality of genomic DNA received, we used the PicoGreen assay and the Qubit 2.0 fluorometer (Invitrogen, Carlsbad, CA, USA). Each sample was quantified in quadruplicate. Samples were pooled at varying ratios to generate the mock community (Supplementary Fig. 1).

Both shotgun sequence datasets were screened for adapters, artifacts, according to quality scores (Illumina: Q12; PacBio: 75%), number of ‘N’, read length (Illumina: min 40 bp, PacBio: min 50 bp), and contaminant sequences related to human, dog, cat, and mouse.

## Additional information

**How to cite**: Singer, E. *et al.* Next generation sequencing data of a defined microbial mock community. *Sci. Data* 3:160081 doi: 10.1038/sdata.2016.81 (2016).

## Supplementary Material

Supplementary information

## Figures and Tables

**Table 1:** Genome statistics of each mock community member.

**Organism**	**Isolation source**	**GenBank Accession ID**	**Genome size [bp]**	**GC [%]**	**% repeats**	**# of scaffolds**	**# of 16S copies**
*Terriglobus roseus* DSM 18391 (AD)	Soil	NC_018014	5227858	60.3	18.3	1	2
*Corynebacterium glutamicum* ATCC 13032 (AT)	Sewage	NC_003450	3309401	53.8	NA*	1	6
*Nocardiopsis dassonvillei* DSM 43111 (AT)	Soil	NC_014211	6543312	72.7	0.2	2	5
*Olsenella uli* DSM 7084 (AT)	Human gingival crevice	NC_014363	2051896	64.7	0.46	1	1
*Segniliparus rotundus* DSM 44985 (AT)	Human sputum	NC_014168	3157527	66.8	0.92	1	1
*Echinicola vietnamensis* DSM 17526 (B)	Seawater collected in a mussel farm	NC_019904	5608040	44.8	4.34	1	4
*Meiothermus Silvanus* DSM 9946 (D)	Hot spring (50 °C)	NC_014212	3721669	62.7	6.54	3	2
*Clostridium perfringens* ATCC 13124 (F)	Bovine	NC_008261	3256683	28.4	2.02	1	20
*Clostridium thermocellum* ATCC 27405 (F)	Various	NC_009012	3843301	39	7.51	1	4
*Desulfosporosinus acidiphilus* SJ4 DSM 22704 (F)	Pond sediment	NC_018068	4991181	42.1	4.08	3	9
*Desulfosporosinus meridiei* DSM 13257 (F)	Aquifer groundwater	NC_018515	4873567	41.8	2.89	1	11
*Desulfotomaculum gibsoniae* DSM 7213 (F)	Freshwater mud	NC_021184	4855529	45.5	5.99	1	8
*Streptococcus pyogenes* M1 GAS SF370 (F)	Infected wound	NC_002737	1852441	38.5	NA*	1	6
*Thermobacillus composti* KWC4, DSM 18247 (F)	Composting reactor	NC_019897	4355525	60.1	7.14	2	5
*Escherichia coli* K-12, MG1655 (P)	Human stool	NC_000913	4639675	50.8	6.7	1	7
*Frateuria aurantia* DSM 6220 (P)	*Lilium auratium*	NC_017033	3603458	63.4	1.32	1	4
*Hirschia baltica* ATCC 49814 (P)	Brackish water	NC_012982	3540114	45.2	0.45	2	2
*Pseudomonas stutzeri* RCH2 (P)	Cr-contaminated aquifer	NC_019936	4600489	62.5	1.83	4	4
*Salmonella bongori* NCTC 12419 (P)	African frog	NC_015761	4460105	51.3	2.36	1	7
*Salmonella enterica subsp. arizonae serovar* RSK2980 (P)	Animal tissue	NC_010067	4600800	51.4	2.42	1	7
*Spirochaeta smaragdinae* DSM 11293 (S)	Oil field	NC_014364	4653970	49	2.01	1	2
*Fervidobacterium pennivorans* DSM 9078 (T)	Hot mud of spa	NC_017095	2166381	39	4.04	1	2
*Coraliomargarita akajimensis* DSM 45221 (V)	Seawater	NC_014008	3750771	53.6	1.07	1	2
*Halovivax ruber* XH-70 (E)	Saline lake	CP003050.1	3223876	64.3	NA*	1	2
*Natronobacterium gregoryi* SP2 (E)	Solar saltworks	NC_019792.1	3788356	62.2	4.22	1	3
*Natronococcus occultus* DSM 3396 (E)	Lake	NC_019974.1	4314118	64.7	0.91	3	4
Genome size includes chromosomes and plasmids. All genomes are available as finished sequences. Phylum associations for each strain are abbreviated as follows: AD—Acidobacteria, AT—Actinobacteria, B—Bacteroidetes, D—Deinococcus-Thermus, E—Euryarchaeota, F—Firmicutes, P—Proteobacteria, S—Spirochaetes, T—Thermotogae, V—Verrucomicrobia. Isolation sources were obtained from literature on respective strains, where available. GC content is based on genome size. Genomes without NCBI repeat region annotation are denoted with an *.							

**Table 2:** Sequence Statistics by sequencing platform.

**Platform**	**Illumina**	**PacBio**
Model	HiSeq-HO 2000	RS II
Library chemistry	TruSeq paired-end cluster kit v3	SMRTbell template preparation kit
Sequencing chemistry	TruSEq SBS sequencing kit 200 cycles v3	P4C2
Run mode	2x150	1x120 min
# of raw reads	355,875,608	300,584
# of filtered reads	347,963,988	53,654
Average insert size [bp]	219±43	1,041±576
Average quality score (filtered reads)	Read 1: 33.47, Read 2: 32.04	0.976
