# Supplementary material for: Next generation sequencing data of a defined microbial mock community
Source: Sci Data. 2016 Sep 27;3:160081. doi: 10.1038/sdata.2016.81 (PMC5037974; doi:10.1038/sdata.2016.81)
Supplement: Supplementary information [file sdata201681-s1.pdf]

Supplementary Information

Table of Contents

Supplementary Table 1……………..…………………………………………………pp. 2-5

Supplementary Figure 1………………………………………………………….. p. 6

Supplementary Figure 2………………………………………………………….. p. 6

Supplementary Figure 3………………………………………………………….. p. 7

Supplementary Table 1 | Detailed molarity and mapping information. Mapped genome includes chromosome and plasmids (if present). Average fold coverages were: 1698X (genome), 516X (chromosome) in the Illumina, and 1.4X (genome), 0.5X (chromosome) in the PacBio shotgun datasets.

Organism	Molarity	Genome copies per µl	Illumina		PacBio		Illumina		PacBio	
			% mapped genome	% mapped chromosome	% mapped genome	% mapped chromosome	% genome covered	% chromosome covered	% genome covered	% chromosome covered
T. roseus	4.79E-15	155	2.07	2.13	4.40	4.23	83.95	83.95	38.16	38.16
C. glutamicum	4.91E-16	10	0.30	0.31	0.47	0.45	99.34	99.34	8.20	8.20
N. dassonvillei	2.67E-17	6	0.00	0.00	0.00	0.00	0.54	0.34	0.00	0.00
O. uli	3.40E-14	304	2.26	2.32	1.64	1.58	100	100	31.39	31.39
S. rotundus	1.22E-14	149	1.41	1.45	2.46	2.36	99.96	99.96	34.88	34.88

E. vietnamensis	1.26E-15	41	0.62	0.64	0.54	0.52	99.29	99.29	5.03	5.03
M. silvanus	4.38E-14	213	8.56	7.82	14.79	12.44	99.97	99.98	88.78	78.92
C. perfringens	5.20E-16	39	0.42	0.43	0.10	0.10	99.53	99.53	1.19	1.19
C. thermocellum	4.40E-16	15	0.43	0.44	0.29	0.28	99.11	99.11	3.83	3.83
D. acidiphilus	2.68E-14	409	15.11	14.75	10.57	9.67	99.98	99.98	61.52	60.31
D. meridiei	9.89E-15	261	4.61	4.74	3.60	3.45	99.74	99.74	29.79	29.79
D. gibsoniae	2.93E-14	535	6.91	7.11	3.89	3.73	99.93	99.93	30.61	30.61
S. pyogenes	1.53E-15	16	0.43	0.44	0.31	0.30	99.06	99.06	7.44	7.44

T. composti	2.39E-16	7	8.50	8.18	12.37	11.45	99.82	99.82	80.43	77.75
E. coli	3.90E-16	16	0.18	0.19	0.26	0.25	98.87	98.87	3.36	3.36
F. aurantia	2.84E-14	317	3.99	4.11	6.22	5.98	99.95	99.95	62.35	62.35
H. baltica	1.78E-14	400	8.16	8.20	8.37	7.85	99.99	99.99	72.45	70.90
P. stutzeri	1.21E-14	164	1.55	1.56	3.32	3.15	99.23	99.23	35.95	35.61
S. bongori	1.72E-16	31	0.14	0.15	0.22	0.21	99.31	99.31	2.85	2.85
S. enterica	6.69E-16	40	0.52	0.54	0.73	0.70	99.63	99.62	8.21	8.21
S. smaragdinae	2.78E-14	467	11.39	11.72	8.96	8.61	100	100	60.42	60.42

F. pennivorans	6.21E-14	672	11.26	11.58	4.50	4.33	100	100	60.17	60.17
C. akajimensis	6.85E-15	144	3.41	3.50	4.96	4.77	99.78	99.78	54.79	54.79
H. ruber	2.34E-14	614	1.75	1.80	1.06	1.02	99.92	99.92	14.08	14.08
N. gregoryi	3.01E-14	569	2.46	2.53	2.77	2.67	99.89	99.89	30.79	30.84
N. occultus	2.15E-14	933	3.55	3.35	3.21	2.88	100	100	32.46	30.44

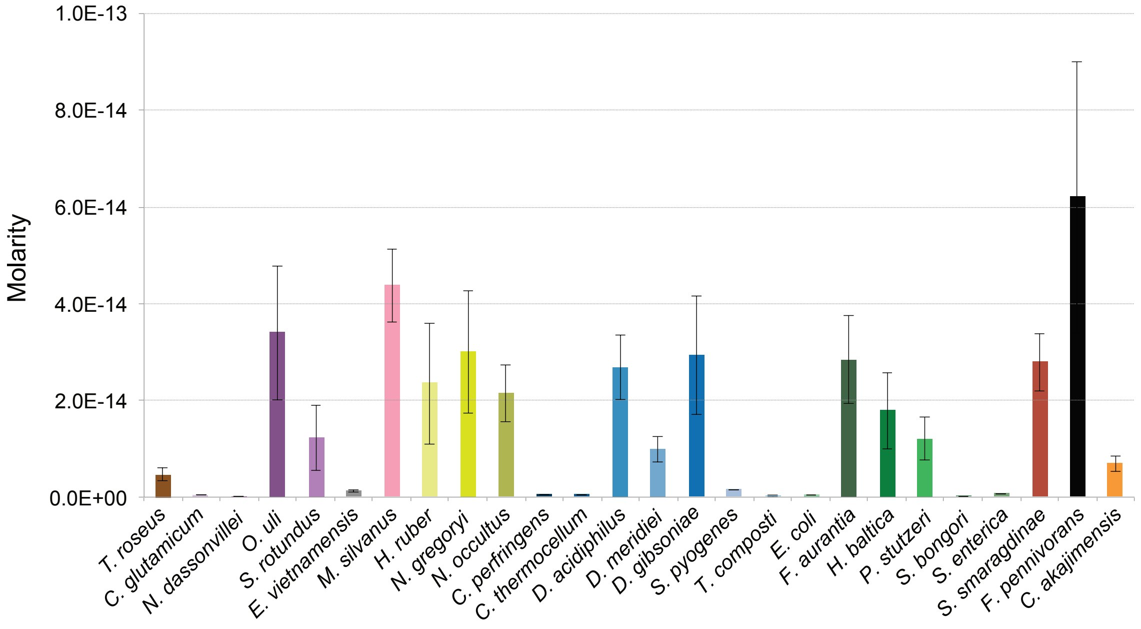

Supplementary Fig. 1 | DNA concentration of each MBARC-26 member used to generate the mock community. Quadruplicate measurements of DNA concentrations using the PicoGreen assay showed large standard deviations for some of the DNA samples. Colors denote phylum association as defined in Fig. 1.

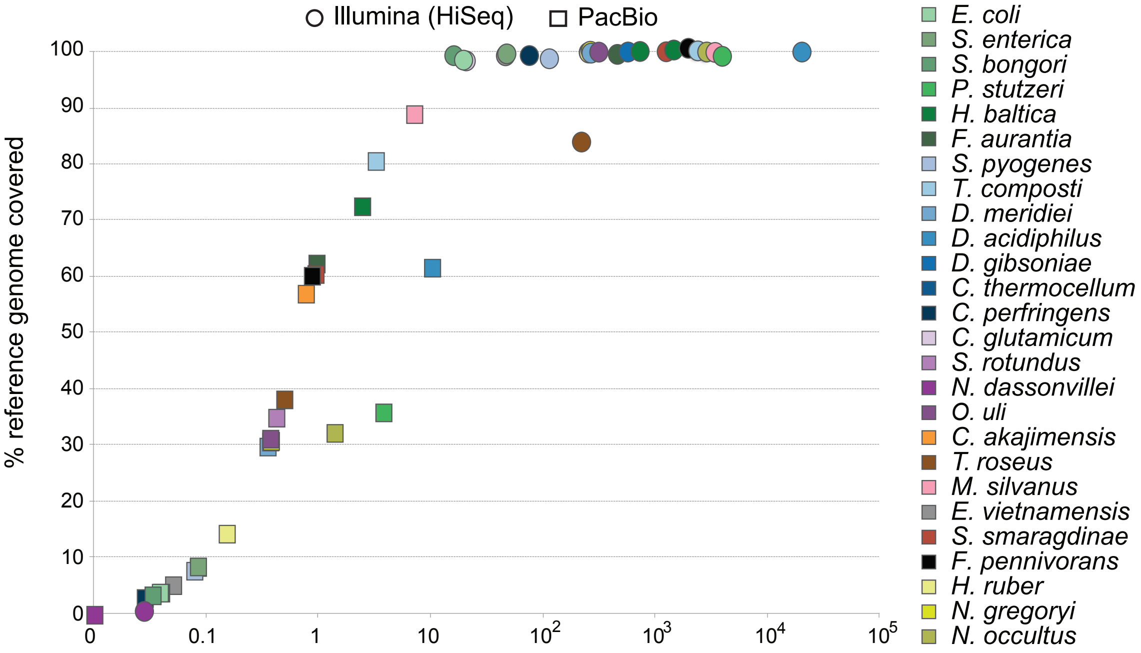

Supplementary Fig. 2 | Percent genome coverage (including plasmids) and fold coverage of each MBARC-26 mock community genome by sequencing platform using unassembled sequences. Colors denote phylum association as defined in Fig. 1.

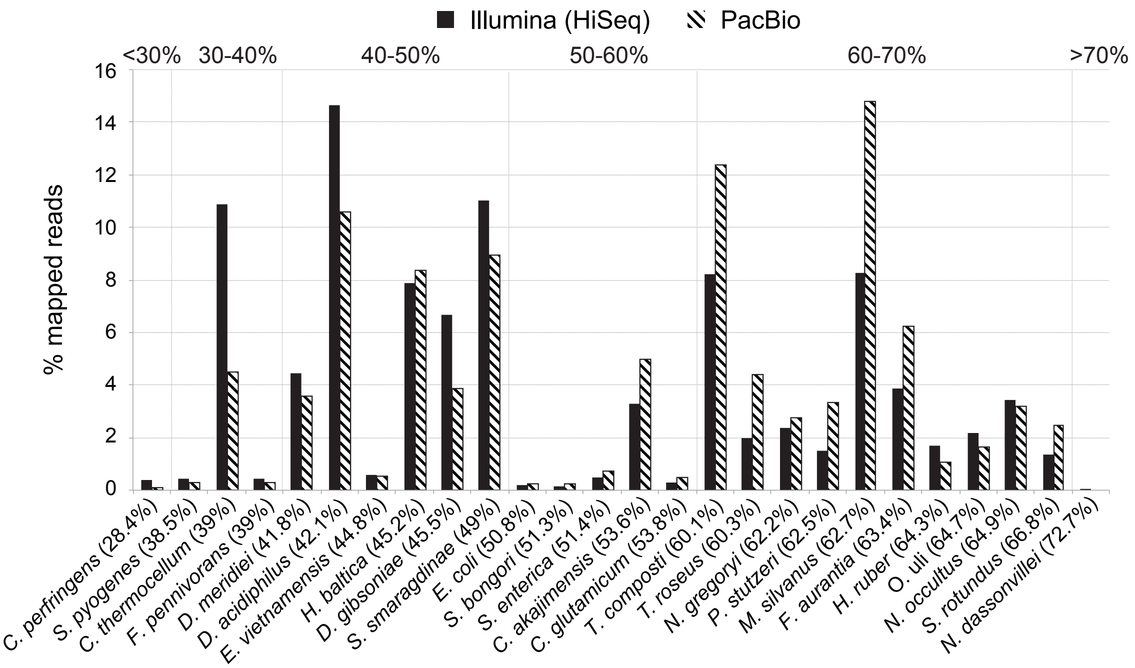

Supplementary Fig. 3 | Percent mapped reads by sequencing platform. MBARC-26 community members are grouped according to their respective GC content. Detailed GC content is displayed in the x-axis and available in Table 1.
